# Brk/PTK6 and Involucrin Expression May Predict Breast Cancer Cell Responses to Vitamin D3

**DOI:** 10.3390/ijms241310757

**Published:** 2023-06-28

**Authors:** Carol Box, Caroline Pennington, Stephen Hare, Sarah Porter, Dylan Edwards, Suzanne Eccles, Mark Crompton, Amanda Harvey

**Affiliations:** 1 The Cancer Research UK Cancer Therapeutics Unit, McElwain Laboratories, The Institute of Cancer Research, Sutton SM2 5NG, UK; carol.box@icr.ac.uk (C.B.); sue.eccles01@icr.ac.uk (S.E.); 2 School of Biological Sciences, University of East Anglia, Norwich NR4 7TJ, UKdylan.edwards@uea.ac.uk (D.E.); 3 Centre for Genome Engineering and Maintenance, Institute for Health Medicine and Environment, Brunel University London, Uxbridge UB8 3PH, UK; stephen.hare1@sky.com; 4 School of Biological Sciences, Royal Holloway University of London, Egham, Surrey TW20 0EX, UK; m.crompton@rhul.ac.uk

**Keywords:** 1,25-dihydroxyvitamin D3, breast cancer, breast tumour kinase (Brk), protein tyrosine kinase 6 (PTK6), differentiation, involucrin

## Abstract

The process of human embryonic mammary development gives rise to the structures in which mammary cells share a developmental lineage with skin epithelial cells such as keratinocytes. As some breast carcinomas have previously been shown to express high levels of involucrin, a marker of keratinocyte differentiation, we hypothesised that some breast tumours may de-differentiate to a keratinocyte-derived ‘evolutionary history’. To confirm our hypothesis, we investigated the frequency of involucrin expression along with that of Brk, a tyrosine kinase expressed in up to 86% of breast carcinomas whose normal expression patterns are restricted to differentiating epithelial cells, most notably those in the skin (keratinocytes) and the gastrointestinal tract. We found that involucrin, a keratinocyte differentiation marker, was expressed in a high proportion (78%) of breast carcinoma samples and cell lines. Interestingly, tumour samples found to express high levels of involucrin were also shown to express Brk. 1,25-dihydroxyvitamin D3, a known differentiation agent and potential anti-cancer agent, decreased proliferation in the breast cancer cell lines that expressed both involucrin and Brk, whereas the Brk/involucrin negative cell lines tested were less susceptible. In addition, responses to 1,25-dihydroxyvitamin D3 were not correlated with vitamin D receptor expression. These data contribute to the growing body of evidence suggesting that cellular responses to 1,25-dihydroxyvitamin D3 are potentially independent of vitamin D receptor status and provide an insight into potential markers, such as Brk and/or involucrin that could predict therapeutic responses to 1,25-dihydroxyvitamin D3.

## 1. Introduction

The Brk tyrosine kinase has been described as a potential therapeutic target for breast cancer treatment since it influences major signalling pathways implicated in tumour development [1]. Previous studies have shown that Brk expression increases anchorage-independent growth and survival [2,3,4,5] and that its suppression by RNA interference reduces the rate of breast cancer cell proliferation in vitro [6]. Brk is expressed in a majority of breast carcinomas [7,8,9]; however, its expression in normal tissues is relatively restricted. Normal expression is limited primarily to cells in differentiating epithelia such as those in the gastrointestinal (GI) tract, where it is present within the non-dividing villus epithelium of the small intestine, as well as in the crypt cells post-irradiation [10,11,12]. Brk expression has also been detected in the nuclei of normal luminal prostate epithelial cells and oral epithelia [13,14].

Brk expression has previously been reported to be low/undetectable in normal human mammary tissue samples, and studies identifying the mouse orthologue, Sik, show that it is not detected at any stage of murine embryonic mammary development [10], although more recent data suggest that Brk is expressed in normal human mammary cells [15].

In human skin, Brk expression is largely restricted to suprabasal keratinocytes in the differentiating layers [16]. Several in vitro studies suggest that Brk/Sik is involved in calcium-induced keratinocyte differentiation, which is characterised by increased expression of epidermal differentiation markers such as Keratin10 or Filaggrin [16,17,18].

The first signs of human mammary gland formation begin early in gestation. They are characterised by the thickening of the ectoderm to form milk streaks, or lines, which then fragment. The thoracic region thickens and widens, while the anterior region begins to involute. A solid bud of cells gives rise to the discrete structures that will form the mammary tissue. The primordium begins to grow down into the underlying mesenchyme and the skin ceases to protrude. Mammary capillaries then start to form and the edge of the anlage forms outgrowths. These outgrowths continue into the mesenchyme and branch to form the epithelial cords that, in turn, give rise to the mammary ducts [19,20].

Ultrastructural studies carried out on week 13 human embryos have shown that, although there was no sign of myoepithelial differentiation in the mammary basal cell layer, cells present at the epithelial–stromal junction sustained the hemidesmosomes and keratin aggregates of skin basal cells. Thus, the embryonic ectoderm gives rise to the epithelia of both the skin and the mammary gland, meaning there are likely to be shared aspects of development.

A high proportion of human breast carcinomas express Brk; however, not all the underlying regulatory mechanisms are known. The expression of Brk in normal skin, coupled with the embryonically-related origins of skin and mammary epithelial cells, has led us to hypothesise that some mammary carcinomas might retain an evolutionary history characteristic of skin epithelial cells. This suggests that the development of some breast tumours might reflect an aberrant differentiation process.

Involucrin is a cytoplasmic protein that becomes cross-linked in terminally differentiating keratinocytes [21,22] enabling its expression to be used as a marker of terminal keratinocyte differentiation. We have previously shown a link between Brk and keratinocyte differentiation markers [17]. This, combined with other studies reporting that around a quarter of breast tumours express involucrin [23,24], implies a possible link between differentiation processes and breast cancer.

We investigated whether Brk and involucrin were co-expressed in breast tumours. In this way, agents that are known to induce differentiation in keratinocytes (such as vitamin D3) may be shown to be more effective in Brk-positive breast cancer cells compared with Brk-negative cells, potentially identifying alternative therapy options.

## 2. Results

**Figure 1:**
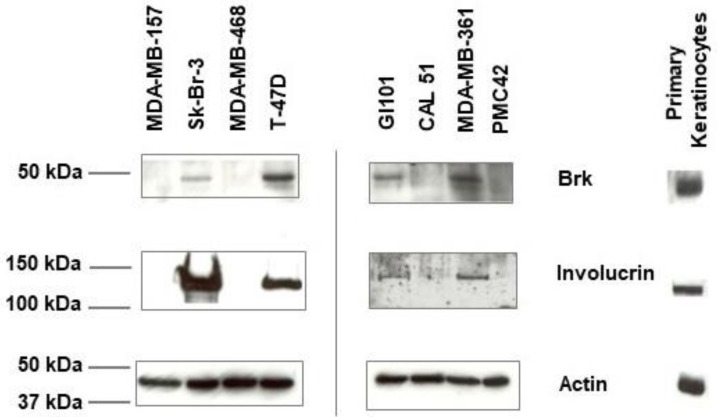
Expression of Brk and involucrin in human breast cancer cell lines. Lysates of the indicated cell lines were prepared from non-confluent proliferating cells and analysed by immunoblotting for Brk (upper panel) and involucrin (middle panel). Loading was verified by blotting for β-actin (lower panel).

To test the hypothesis that Brk-positive breast tumour cells have a partial keratinocyte-like phenotype, whole cell lysates generated from eight breast cancer cell lines were analysed by Western blotting for the presence of Brk and involucrin (Figure 1). The cell lines were selected for their known Brk status; four were positive and four were negative, based on previous findings [4,7]. The Brk-positive cell lines, T-47D, MDA-MB-361, GI101 and SkBr3 had detectable levels of involucrin protein. The four Brk-negative cell lines, MDA-MB157, MDA-MB468, Cal51 and PMC42, showed no detectable involucrin expression. These results indicate that Brk and involucrin may be co-expressed in breast cancer cell lines.

**Figure 2:**
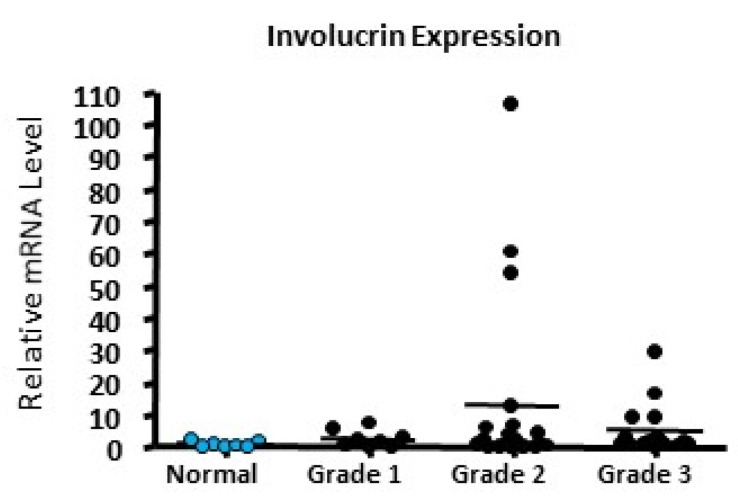
Analysis of involucrin mRNA expression in human breast carcinomas. Quantitative RT-PCR was carried out on mRNA extracted from normal and tumour samples (Grades 1, 2 and 3). Relative levels of involucrin mRNA expression are plotted against histological grade; 34 out of 44 samples showed elevated expression of involucrin expression (determined by subtracting the median expression levels of the normal breast samples from the tumour samples).

To establish whether this finding was an artefact of cultured cell lines and, therefore, not representative of tumours in vivo, quantitative RT-PCR was carried out using mRNA extracted from 44 patient surgical tumour samples of known grade as well as 7 normal breast tissue samples (Figure 2). Surprisingly, we were able to detect involucrin expression in our samples. The normal mammary samples were found to express the lowest levels of involucrin mRNA. All 46 tumour samples assessed were initially positive for involucrin expression; however, when we subtracted the median expression level of the normal samples, 36 out of the 46 (78%) tumour samples analysed expressed elevated levels of involucrin compared to normal tissues. Median relative expressions levels were 0.296 (for normal tissues) and 1.658, 1.335 and 1.351 in samples from Grades 1, 2 and 3 carcinomas (respectively), indicating that a small number of outliers with high expression (Figure 2) are skewing the overall mathematical mean expression levels, although the differences between grades did not reach statistical significance.

The 17 tumour samples that expressed the highest levels of involucrin (ten-fold or more than the median level expressed by the normal samples), were all found to be positive for Brk mRNA expression. Five out of the 6 samples (83%) that expressed between five- and ten-fold more involucrin than the normal samples were also Brk positive (Table 1). Brk expression in these tumours has been analysed previously and shown to correlate with tumour grade [4]. While there is no direct correlation between absolute levels of Brk and involucrin mRNA expression, it would appear that Brk is expressed in higher-grade involucrin-positive tumours. These data lend support to the hypothesis that some Brk-positive breast cancers may share features of differentiating keratinocytes and, while not co-dependent, Brk and involucrin expression may be co-regulated in some cases.

**Figure 3:**
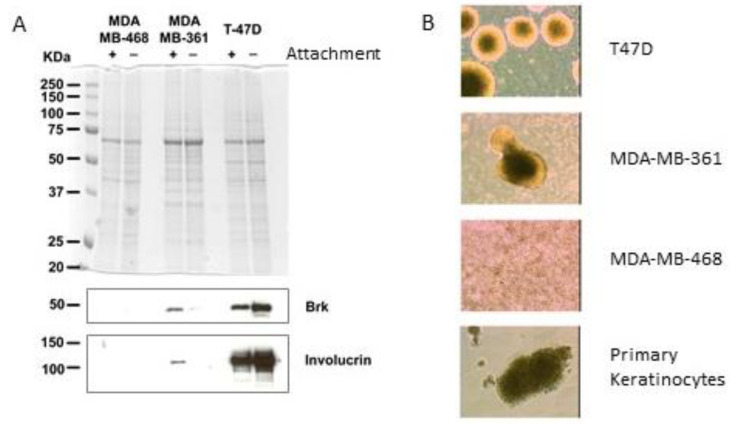
Suspension culture affects expression of differentiation markers. (**A**) Breast cancer cell lines were cultured in the presence or absence of attachment (+/−) on plastic (+) or polyHEMA (−) for 7 days (as +/− attachment) and lysed in SDS sample buffer. SDS-PAGE was carried out followed by Coomassie staining to confirm equal loading (upper panel) and lysates were subsequently analysed for the presence of keratinocyte markers by immunoblotting (lower panel). (**B**) Morphology of cells in suspension culture after 7 days (magnification ×40).

Keratinocytes are known to differentiate when cultured in suspension [25,26], and both Brk and involucrin are co-regulated during keratinocyte differentiation [17]. Therefore, the effect of culturing breast cancer cells in suspension on Brk and involucrin expression was examined. T-47D and MDA-MB-361 were selected based on their medium Brk and involucrin expression levels (compared to the other cell lines shown in Figure 1). This meant that any changes in protein levels (up or down) could be visualised. Western blotting of whole cell lysates showed that involucrin levels were increased in T-47D and decreased in MDA-MB-361 after 7 days of growth in suspension in polyHEMA-coated wells compared with adherent cells grown as a monolayer (Figure 3A). The levels of Brk expression also differed between the adherent and suspension cultures and mirrored involucrin levels, again suggesting that Brk and involucrin may be co-regulated. Interestingly, the Brk/involucrin positive cells also exhibited characteristics of the primary keratinocytes by forming dense spheres in suspension culture that could not be disaggregated upon pipetting. Conversely, the Brk-negative MDA-MB-468 cells remained as single cells in suspension (Figure 3B).

**Figure 4:**
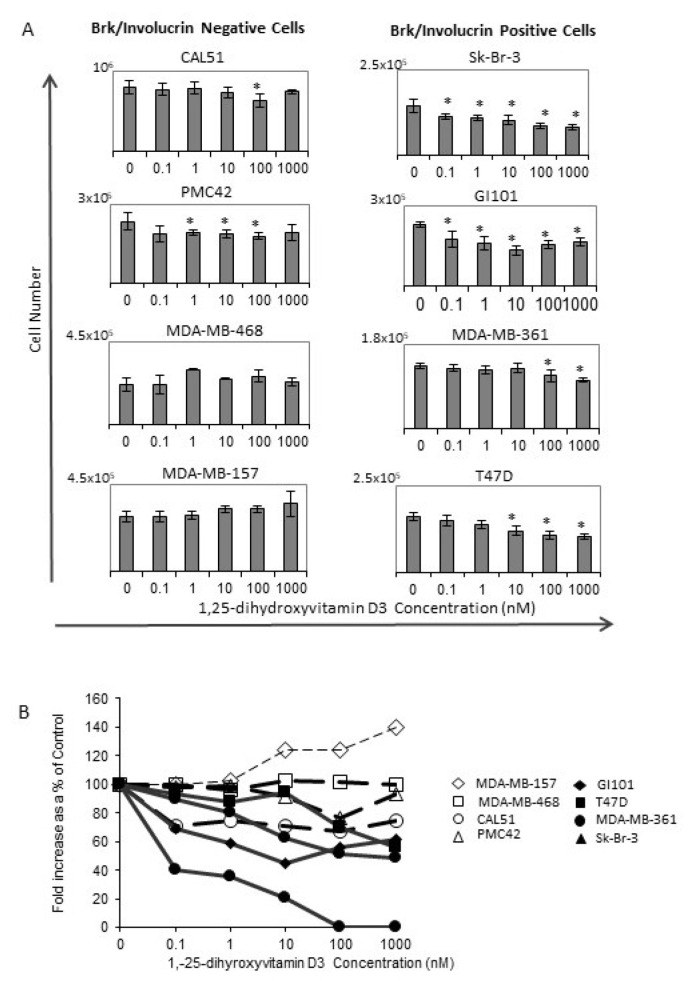
Effect of Vitamin D3 treatment on breast cancer cell proliferation. (**A**) Human breast cancer cells were seeded into 24-well plates, allowed to adhere and then treated with 1,25-dihydroxyvitamin D3 at the concentrations indicated. Viable cell numbers were determined by counting trypan blue-excluding cells in a haemocytometer after 72 h. The mean viable cell numbers +/− SD are plotted (* *p* < 0.05). (**B**) The fold increase in cell number over the 72 h from the day of treatment t = 0 was calculated and expressed as a % of the vehicle-treated controls.

If Brk expression is related to differentiation capacity, especially to a keratinocyte-like phenotype, we hypothesised that Brk-positive breast cancer cells may be susceptible to growth inhibition by keratinocyte-differentiating agents such as vitamin D3. Therefore 1,25-dihydroxyvitamin D3 (calcitriol), the biologically active metabolite of vitamin D3, was tested on cultures of breast cancer cell lines. Cells were treated with 1,25-dihydroxyvitamin D3 and counted after 72 h. The most consistent responses were observed in the cell lines that were positive for both Brk and involucrin expression (Figure 4A).

**Figure 5:**
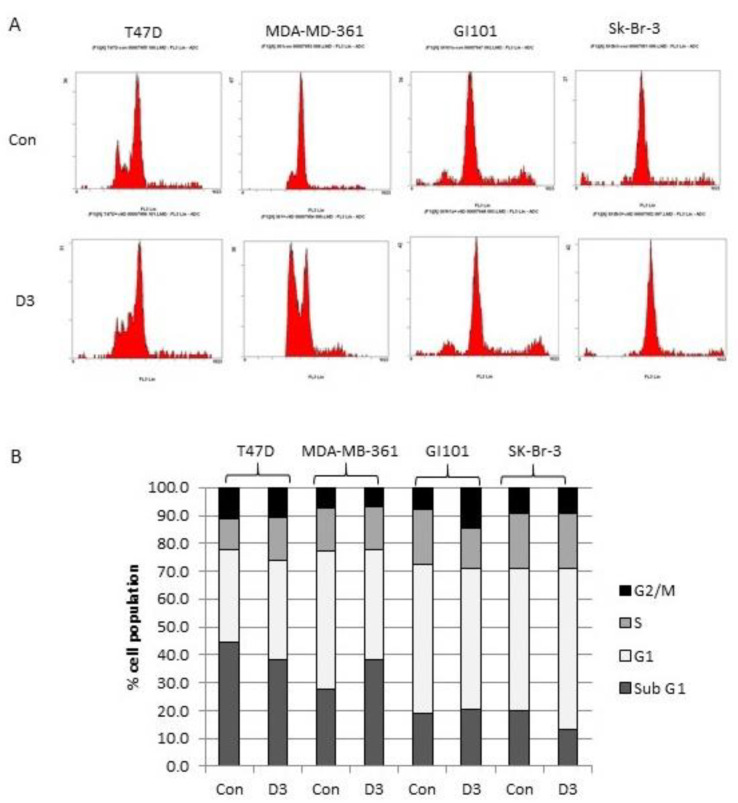
Effect of Vitamin D3 treatment on cell cycle profile. The Brk/involucrin positive cells were seeded, allowed to adhere and then treated with vehicle [0.1% (*v*/*v*) ethanol] (Con) or 100 nM 1,25-dihydroxyvitamin D3 (D3) for 72 h. Cells were subsequently harvested and analysed for cell cycle status by flow cytometry. (**A**) Flow cytometry charts are shown with cell counts on the *y*-axis and FL-3 Lin on the *x*-axis and (**B**) the percentage of cells in each phase of the cell cycle was determined.

The fold increase in cell number over the 72 h period was also determined (and expressed as a percentage of the vehicle control) to compare the effects between the different cell lines more clearly. The four Brk/involucrin-positive cell lines showed smaller increases in total cell number compared with the four Brk/involucrin-negative cell lines (Figure 4B). Interestingly, the cell line with the highest involucrin expression (Sk-Br-3) (Figure 1) had the most marked response to 1,25-dihydroxyvitamin D3 treatment, suggesting that involucrin might be a greater influencing factor in Vitamin D responses than Brk. The decreases in viable cell numbers in the Brk/involucrin positive cells compared with the untreated controls were not accompanied by any consistent alterations in the cell cycle profile (Figure 5). There was not a consistent increase in cell death across all 4 cell lines tested in response to 1,25-dihydroxyvitamin D3 (determined by flow cytometry and based on the absence of an increased sub-G0/G1 peak).

**Figure 6:**
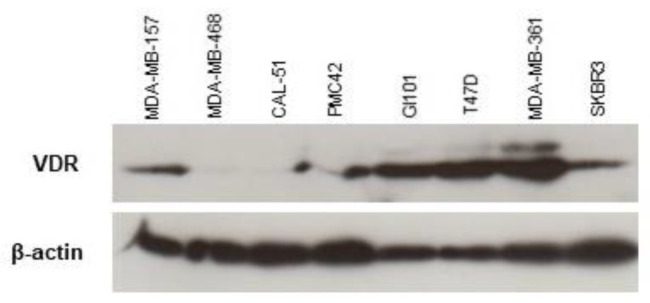
VDR expression does not predict response to 1,25-dihydroxyvitamin D3. Cell lysates were prepared and analysed for VDR expression by Western blotting. The lysates were loaded onto the gel in order of 1,25-dihydroxyvitamin D3 responsiveness (based on the data presented in Figure 4).

Finally, we examined whether responses to 1,25-dihydroxyvitamin D3 correlated with vitamin D eeceptor (VDR) expression. There was no direct correlation between cell responsiveness to 1,25-dihydroxyvitamin D3 and VDR levels (Figure 6).

## 3. Discussion

Mammary tissue is formed from invaginations of the ectoderm and, as differentiating skin cells express Brk, this study attempted to address whether Brk-positive breast tumours had retained/regained some of this previous developmental keratinocyte-like differentiation capacity.

In the human breast cancer cell lines we studied, all of the Brk-positive cultures also expressed detectable levels of involucrin. A total of 77% of the clinical cancer samples were involucrin positive, a much higher proportion than previously reported [23,24], possibly due to the increased sensitivity of RT-PCR in comparison with immunohistochemistry, although it remains a possibility that a subset of tumours expresses involucrin mRNA that is not translated. Tsuda and colleagues showed that involucrin expression correlated with the presence of squamoid features and noted that involucrin expression was associated with higher-grade atypia [24].

In this study, we have shown that involucrin expression can be detected in all tumour grades and that expression levels increased with tumour grade (although the increases did not reach statistical significance Student’s *t*-test *p* > 0.05). In addition, all the tumours that highly overexpressed involucrin also expressed Brk, suggesting that some Brk-positive breast cancers may have a more differentiated keratinocyte-like (or squamoid) phenotype at a cellular level.

Brk expression increases with tumour grade and we have previously suggested that Brk may function in the development of metastatic disease by increasing cell survival in suspension. This would allow tumour cells to survive during dissemination [4]. When keratinocytes begin to differentiate, they migrate away from the basal layer and basement membrane, thereby losing contact with some extracellular matrix proteins. It is possible that acquiring Brk expression during the differentiation process protects cells from cell death on the loss of anchorage and facilitates cell survival until terminal differentiation and cornification commence.

Treatment of keratinocytes with vitamin D or its analogues results in suppressed proliferation and induced differentiation [27,28]. Evidence is accumulating to suggest that vitamin D3 derivatives may have potential benefits as tumour therapies [29], and some agents have been shown to be tolerated and have activity in clinical trials [30,31].

1,25-dihydroxyvitamin D3, the biologically active and far more potent metabolite of vitamin D3 [32], has previously been shown to decrease proliferation [33,34,35] and cause cell cycle arrest at the G1/S-phase transition [36,37]. Along with its analogues, it induces a more differentiated phenotype as determined by lipid droplet formation in MCF7 and MDA-MD-231 breast cancer cell lines at concentrations between 10^−10^ and 10^−6^ M [38]. In our study, the presence of Brk and involucrin appeared to correlate with whether 1,25-dihydroxyvitamin D3 was able to decrease cell proliferation. The Brk-positive cell lines showed reduced cell numbers relative to controls, whereas the Brk-negative breast cancer cell lines were less affected by the doses used (range of 10 nM to 10 μM). Three of the Brk-negative cell lines studied (MDA-MB-468, MDA-MB-157 and CAL-51) were unaffected by these concentrations of 1,25-dihydroxyvitamin D3. Whilst the presence of Brk and involucrin may influence responses to 1,25-dihydroxyvitamin D3, the most marked response was in the Sk-Br-3 cell line which had the most involucrin, suggesting that involucrin levels may play a greater role than those of Brk. Dissecting this will be a focus of ongoing work.

Previous studies have shown that vitamin D3 analogues have more potent anti-proliferative effects in transformed cells compared with their non-transformed counterparts and that hormone sensitivity may play a role. Proliferation and cell cycle progression of untransformed MCF12F cells were unaffected by vitamin D3 analogues; however, the transformed sub-lines MCF12F-DMBA and -NMU were sensitive to both 1,25-dihydroxyvitamin D3 and its analogue 1α-hydroxyvitamin D5 [34]. This study also reported that the analogue had no effect on the oestrogen receptor (ER) negative cell line MDA-MB-231, but did in ER-positive cell lines BT474 and MCF7 [34], despite the fact that MDA-MB-231 is positive for the vitamin D receptor (VDR) [35]. Our data also support the notion that there are cell responses to vitamins that are independent of VDR expression (Table 2).

Breast cancer cell lines were analyzed for VDR, Brk and involucrin expression (Figure 1 and Figure 6) by Western blotting. + = positive expression; − = low/negative expression. ER positivity is based on the published literature [39,40,41].

More recent evidence suggests that VDR expression is linked to that of the ER [42] and that vitamin D responses may also be mediated independently of the VDR in ER-positive cell lines [33]. Given that both MDA-MB-468 (non-responsive to 1,25-dihydroxyvitamin D3, Figure 4) and Sk-Br-3 (the strongest responding cell line, Figure 4) have similar levels of the receptor for 1,25-dihydroxyvitamin D3 (VDR) [43], our data add support to the idea that responses may be mediated separately from receptor expression. As these cell lines are also ER-negative [44], it would appear that the suggestion posed by Lopes and colleagues (2010) is not limited to ER-positive cell lines [42]. It is worth noting, however, that, of the eight breast cancer cell lines we examined, the ER-negative Sk-Br-3 had the highest level of involucrin expression which could be an indicator of responsiveness irrespective of ER status.

Unlike some previous studies in breast cells, we found that although cell proliferation was reduced, there was little change in the cell cycle profile. A similar phenomenon was also observed in U937 leukaemia cells where 100 nM 1,25-dihydroxyvitamin D3 caused a decrease in colony formation but had minimal effects on the cell cycle stage [39]. This suggests that decreased rates of proliferation could occur due to a more uniform decrease in cell cycle progression rather than an accumulation of cells in a particular phase.

We have previously reported that Brk is a potential target for anti-proliferative therapy [6,45]. An alternative approach to preventing proliferation could be to induce keratinocyte-like differentiation with agents such as 1,25-dihydroxyvitamin D3, thereby preventing disseminating cells from dividing. Clinical data would support this approach as up to 70% of women diagnosed with breast cancer are vitamin D3 deficient [46]. Clinical trials with vitamin D supplementation as a therapeutic agent are currently ongoing in breast cancer. Results from prostate cancer patients show potential with patients treated with a combination of high-dose oral 1,25-dihydroxyvitamin D3 and docetaxel having a significantly improved overall survival compared to those treated with docetaxel alone [47].

The data presented in this study suggest that cells that are positive for Brk and involucrin might respond more readily to differentiation-inducing agents, such as vitamin D, than those that do not express Brk or involucrin. They contribute to growing knowledge enabling more precise predictions of cellular responses to 1,25-dihydroxyvitamin D3 by identifying biomarkers that are potentially independent of vitamin D receptor status.

## 4. Materials and Methods

### 4.1. Cell Culture

Human breast cancer cell lines MDA-MB-361 (luminal), MDA-MB-468 (Basal), MDA-MB-157 (Basal B), T47D (luminal) and Sk-Br-3 (luminal), were all purchased from ATCC (Catalogue HTB-27 HTB-132, HTB-24, HTB-133, HTB-30, respectively); PMC42 (Basal) were obtained from Michael O’Hare and CAL51 (Basal) were obtained from DSMZ (ACC302). They were all cultured in RPMI-1640 medium supplemented with 10% foetal bovine serum, 2 mM L-glutamine, 100 U/mL penicillin and 100 µg/mL streptomycin (all Invitrogen) at 37 °C in a humidified atmosphere of 5% CO_2_ in the air. GI101 (Basal) were acquired through our collaboration with the Eccles laboratory and cultured in the same medium with the additional supplement of 5 µg/mL insulin. Cells were sub-cultured twice weekly.

### 4.2. Vitamin D Treatment

Cells were seeded at 50,000 cells per well into 24-well plates and allowed to adhere for 24 h. Cell numbers were determined (T = 0) by haemocytometer counting of replicate wells and the medium was removed from remaining wells and substituted with fresh medium containing either 1,25-dihydroxyvitamin D3 (SelleckChem, Planegg, Germany) at the indicated concentrations or 100% ethanol diluted to 0.1% in culture medium as vehicle control. After a further 72 h, cell numbers were counted. The fold increase in cell number from T = 0 was then calculated.

### 4.3. Breast Carcinoma Samples

Human breast tumour samples: Forty-eight women aged 40–88 years (median age 58 years) provided samples of 50 primary breast neoplasms after informed consent. Two patients who had bilateral disease gave two samples, one from each side. Forty-eight of the neoplasms were invasive carcinomas, and two were high-grade ductal carcinoma in situ, one of which showed foci of micro-invasion. All samples were taken at surgery. The specimens were sent immediately after removal to the laboratory, where, after inking the margins and slicing the specimen, the histopathologist removed a small part of the tumour. Specimens were prepared and snap-frozen in the Norfolk and Norwich University Hospital’s tumour tissue bank as described previously [48]. The rest of the surgical specimen was fixed in 10% formol saline and processed for routine histopathological diagnosis and establishment of prognostic factors.

Human normal mammary tissue: Samples of normal mammary tissue were obtained from 7 patients with informed consent at reduction mammoplasty. All samples were snap-frozen and stored as described for the cancers [48]. Samples were collected with the approval of the Norwich District Ethics Committee, the Norwich Research and Development Committee and the Partners in Cancer Research Tissue Bank Committee.

### 4.4. RNA Extraction

RNA was extracted from 44 of the frozen human breast tumour surgical samples for which histological grade had been determined, and from the seven normal human mammary tissue samples. Total RNA was isolated using a modification of the SV Total RNA Isolation System (Promega, Madison, WI, USA) [49]: 50–100 mg of tumour tissue were homogenized in 1 mL of RNazol B reagent (Biogenesis Ltd., Poole, UK) using an UltraTurrax T8 homogenizer (IKA). The homogenate was stored at −80 °C pending the following step of the protocol: the samples were allowed to thaw completely at room temperature, centrifuged at 14,000 rpm for 10 min and the clear supernatants collected into 200 µL of chloroform. They were then shaken vigorously for 15 s, incubated for 3 min at room temperature and centrifuged for 15 min at 14,000 rpm. The upper phase was collected into 200 µL of 95% ethanol, mixed and transferred into a spin basket assembly. Then, the Promega protocol was followed from step 7 to the end according to the manufacturer’s instructions.

### 4.5. Reverse Transcription

One µg of total RNA was primed with 0.5 µg of random hexameric primers (Promega) and reverse transcribed into cDNA in a 20 µL reaction volume containing 200 units of SuperScript II reverse transcriptase, 5 × first-strand buffer [final concentrations: 50 mM Tris-HCl (pH8.3), 75 mM KCl, and 3 mM MgCl_2_], 10 mM DTT, 0.5 mM of each deoxynucleotide triphosphate (dATP, dCTP, dGTP, and dTTP; Life Technologies, Inc., Carlsbad, CA, USA), and 40 units of RNasin RNase inhibitor (Promega, Southampton, UK). Each priming reaction was carried out at 70 °C for 10 min and stopped by placing samples on ice; the reverse transcription was carried out at 42 °C for 1 h followed by incubation at 70 °C for 10 min.

### 4.6. TaqMan Real-Time PCR

Forward and reverse primers and fluorescence-labelled oligonucleotide probes (using 6-carboxy-fluorescein (FAM) as the reporter dye and 6-carboxytetramethylrhodamine (TAMRA) as the quencher dye) were designed for the human *Brk/PTK6* gene using Primer Express 1.0 software (PE Applied Biosystems, Foster City, CA, USA) and synthesized by PE Applied Biosystems (Warrington, UK). BLASTN searches were undertaken on our designed primers to confirm gene specificity. To ensure against genomic DNA amplification, the forward and reverse primers were placed in separate adjacent exons. A standard curve with concentrations ranging from 25 ng to 1 ng was produced using human placental cDNA dissected from foetal villi as the template. An XY scatter plot for each gene was produced using Microsoft Excel Chart Wizard software (Microsoft Office 2001), and values for the equation *y* = *mx* + *b* (where *m* = the slope of the standard curve and *b* = the *y* intercept of that line) and R2 were obtained. The *18S* rRNA gene was used as an endogenous control to normalize for differences in the amount of total RNA in each sample. PCR reactions for all samples were performed in duplicate in 96-well optical plates with 5 ng of cDNA (1 ng of cDNA for the *18S* gene), 100 nM probe, 200 nM each primer and 12.5 µL of TaqMan Universal 2 × PCR Master Mix (PE Applied Biosystems, Warrington, UK) in a 25 µL reaction volume. The amplification reaction was carried out over 40 cycles with the following parameters: an initial holding stage of 2 min at 50 °C and then 10 min at 95 °C, followed by a two-step cycling program of 15 s at 95 °C and 1 min at 60 °C.

### 4.7. Western Blotting

Cells for analysis were lysed in 2 × SDS-PAGE lysis buffer and proteins were separated by SDS-polyacrylamide gel electrophoresis. After electro-transfer in Towbin buffer (2.5 mM Tris/19.2 mM Glycine/0.1% SDS/20% methanol), membranes were blocked in 5% non-fat milk protein in TBS/0.1% Tween (TBS-T). Membranes were then incubated at 4 °C overnight with primary antibody to involucrin (Sigma-Aldrich, Gillingham, UK), VDR (Cell Signaling Technology, Leiden, The Netherlands), β-Actin (Abcam, Cambridge, UK) or GAPDH (Abcam) in 5% non-fat milk protein/TBS-T; or to Brk (ICR-100), in 5% BSA/TBS-T. Proteins were visualised with an appropriate hrp-conjugated secondary antibody (Dako, via Agilent, Stockport, UK) and chemiluminescent substrate (Pierce, via Thermofisher, Loughborough, UK).

### 4.8. Flow Cytometry

Cells treated with either 100 nM 1,25-dihydroxyvitamin D3 or 0.1% (*v/v*) ethanol (controls) for 72 h were detached, counted and resuspended at 10^6^/mL in 70% (*v/v*) ethanol. After overnight incubation at 4 °C, cells were centrifuged at 550× *g* for 5 min. Cells were then resuspended in PBS containing 4 µg/mL propidium iodide and 10 µg/mL ribonuclease A and incubated at 37 °C for 45 min, then 4 °C overnight. Samples were analysed on a Beckman Coulter FC500 flow cytometer.

### 4.9. Suspension Culture

Tissue culture plates were coated with a volume of poly(2-hydroxyethylmethacrylate) (polyHEMA, Sigma, Poole, UK) in 98% ethanol (20 mg/mL) to give a final polyHEMA concentration of 1.5 mg/cm^2^. Plates were air-dried in a tissue culture cabinet [4]. Cells were seeded into polyHEMA-coated wells at 0.75 × 104 cells/cm^2^. After 7 days in suspension culture, cells were harvested and lysed in 2 × SDS-PAGE lysis buffer.

## Figures and Tables

**Table 1:** Relationship between Brk and involucrin mRNA expression in breast tumour samples.

Tumour ID	Involucrin Expression	Normalised Involucrin Expression Level	Fold Increase in Involucrin Expression	Involucrin Expression Level	Brk Expression	Normalised Brk Expression Level	Fold Increase in Brk Expression	Brk Expression Level
915	N				Y	1.589	10	HIGH
911	N				Y	5.367	33	HIGH
925	N				Y	4.112	25	HIGH
917	N				Y	0.577	4	MED
905	N				Y	2.14	13	HIGH
906	N				N			
922	N				Y	3.455	21	HIGH
904	N				N			
909	N				Y	1.162	7	MED
907	N				Y	5.829	36	HIGH
910	Y	0.029	0	LOW	Y	15.457	94	HIGH
834	Y	0.046	0	LOW	N			
814	Y	0.132	0	LOW	Y	0.701	4	LOW
836	Y	0.174	1	LOW	Y	0.94	6	LOW
916	Y	0.175	1	LOW	Y	2.031	12	HIGH
843	Y	0.222	1	LOW	Y	0.127	1	LOW
817	Y	0.352	1	LOW	Y	3.462	21	HIGH
903	Y	0.542	2	LOW	Y	1.621	10	HIGH
927	Y	0.607	2	LOW	Y	1.79	11	HIGH
841	Y	0.631	2	LOW	N			
901	Y	0.855	3	LOW	Y	2.074	13	HIGH
805	Y	0.984	3	LOW	Y	3.987	24	HIGH
919	Y	1.126	4	LOW	Y	8.411	51	HIGH
920	Y	1.361	5	MED	Y	9.865	60	HIGH
827	Y	1.447	5	MED	Y	4.078	25	HIGH
844	Y	1.475	5	MED	Y	0.619	4	LOW
810	Y	1.87	6	MED	Y	2.171	13	HIGH
824	Y	2.224	8	MED	N			
801	Y	2.62	9	MED	Y	2.88	18	HIGH
812	Y	2.845	10	HIGH	Y	4.67	28	HIGH
924	Y	3.406	12	HIGH	Y	1.698	10	HIGH
926	Y	3.746	13	HIGH	Y	13.511	82	HIGH
815	Y	5.355	18	HIGH	Y	1.596	10	HIGH
802	Y	5.477	19	HIGH	Y	5.191	32	HIGH
908	Y	6.25	21	HIGH	Y	3.312	20	HIGH
818	Y	6.943	23	HIGH	Y	1.194	7	MED
804	Y	7.361	25	HIGH	Y	0.341	2	LOW
914	Y	8.636	29	HIGH	Y	2.485	15	HIGH
921	Y	8.636	29	HIGH	Y	5.637	34	HIGH
820	Y	12.266	41	HIGH	Y	0.74	5	MED
845	Y	16.048	54	HIGH	Y	2.394	15	HIGH
923	y	28.93	98	HIGH	Y	9.537	58	HIGH
902	Y	53.315	180	HIGH	Y	1.71	10	HIGH
913	Y	55.088	186	HIGH	Y	2.008	12	HIGH
831	Y	60.149	203	HIGH	Y	4.519	28	HIGH
807	Y	105.604	357	HIGH	Y	0.753	5	MED

Tumour samples were analysed for involucrin and Brk expression (indicated by Y/N) and fold increase in gene expression compared to the normal samples was calculated. Low expression was defined as <5 times the expression of the normal tissue and medium (MED) expression as >5 but <10 times the expression of the normal.

**Table 2:** Expression of ER, VDR, Brk and Involucrin in breast cancer cell lines.

	MDA- MB- 157	MDA- MB- 468	CAL 51	PMC 42	GI 101	T47D	MDA- MB- 361	SK-BR-3
ER	−	−	−	+	+/−	+	+	−
VDR	+	−	−	+	+	+	+	+
Brk	−	−	−	−	+	+	+	+
Involucrin	−	−	−	−	+	+	+	+

## Data Availability

No datasets were generated during this study.

## References

[1] Harvey A.J., Burmi R.S., Gunduz E., Gundez M. (2011). Future therapeutic strategies: Implications for Brk targeting. Breast Cancer—Current and Alternative Therapeutic Modalities.

[2] Kamalati T., Jolin H.E., Mitchell P.J., Barker K.T., Jackson L.E., Dean C.J., Page M.J., Gusterson B.A., Crompton M.R. (1996). Brk, a Breast Tumor-derived Non-receptor Protein-tyrosine Kinase, Sensitizes Mammary Epithelial Cells to Epidermal Growth Factor. J. Biol. Chem..

[3] Kim H.I., Lee S.-T. (2005). An Intramolecular Interaction between SH2-Kinase Linker and Kinase Domain Is Essential for the Catalytic Activity of Protein-tyrosine Kinase-6. J. Biol. Chem..

[4] Harvey A.J., Pennington C.J., Porter S., Burmi R.S., Edwards D.R., Court W., Eccles S.A., Crompton M.R. (2009). Brk Protects Breast Cancer Cells from Autophagic Cell Death Induced by Loss of Anchorage. Am. J. Pathol..

[5] Irie H.Y., Shrestha Y., Selfors L., Frye F., Iida N., Wang Z., Zou L., Yao J., Lu Y., Epstein C.B. (2010). PTK6 Regulates IGF-1-Induced Anchorage-Independent Survival. PLoS ONE.

[6] Harvey A.J., Crompton M.R. (2003). Use of RNA interference to validate Brk as a novel therapeutic target in breast cancer: Brk promotes breast carcinoma cell proliferation. Oncogene.

[7] Barker K.T., Jackson L.E., Crompton M.R. (1997). BRK tyrosine kinase expression in a high proportion of human breast carcinomas. Oncogene.

[8] Zhao CYasui K., Lee C.J., Kurioka H., Hosokawa Y., Oka T., Inazawa J. (2003). Elevated Expression Levels of NCOA3, TOP1, and TFAP2C in Breast Tumors as Predictors of Poor Prognosis. Cancer.

[9] Born M., Quintanilla-Fend L., Braselmann H., Reich U., Richter M., Hutzler P., Aubele M. (2005). Simultaneous over-expression of the Her2/neu and PTK6 tyrosine kinases in archival invasive ductal breast carcinomas. J. Pathol..

[10] Llor X., Serfas M.S., Bie W., Vasioukhin V., Polonskaia M., Derry J., Abbott C., Tyner A. (1999). BRK/Sik expression in the gastrointestinal tract and in colon tumors. Clin. Cancer Res..

[11] Vasioukhin V., Serfas M.S., Siyanova E.Y., Polonskaia M., Costigan V.J., Liu B., Thomason A., Tyner A. (1995). A novel intracellular epithelial cell tyrosine kinase is expressed in the skin and gastrointestinal tract. Oncogene.

[12] Haegebarth A., Perekatt A.O., Bie W., Gierut J.J., Tyner A.L. (2009). Induction of Protein Tyrosine Kinase 6 in Mouse Intestinal Crypt Epithelial Cells Promotes DNA Damage–Induced Apoptosis. Gastroenterology.

[13] Derry J.J., Prins G.S., Ray V., Tyner A.L. (2003). Altered localization and activity of the intracellular tyrosine kinase Brk/sik in prostate tumour cells. Oncogene.

[14] Petro B., Tan R., Tyner A., Lingen M., Watanabe K. (2004). Differential expression of the non-receptor tyrosine kinase BRK in oral squamous cell carcinoma and normal oral epithelium. Oral Oncol..

[15] Peng X., Hawthorne M., Vaishnav A., St-Arnaud R., Mehta R.G. (2009). 25-hydroxyvitamin D_3_ is a natural chemopreventative agent against carcinogen induced precancerous lesions in mouse mammary gland organ culture. Breast Cancer Res. Treat..

[16] Wang T., Jee S., Tsai T., Huang Y., Tsai W., Chen R. (2005). Role of breast tumour kinase in the in vitro differentiation of HaCaT cells. Br. J. Dermatol..

[17] Tupper J., Crompton M.R., Harvey A.J. (2011). Breast tumor kinase (Brk/PTK6) plays a role in the differentiation of primary keratinocytes. Arch. Dermatol. Res..

[18] Vasioukhin V., Tyner A.L. (1997). A role for the epithelial-cell-specific tyrosine kinase Sik during keratinocyte differentiation. Proc. Natl. Acad. Sci. USA.

[19] Mikkola M.L., Miller S.E. (2006). The Mammary Bud as a Skin Appendage: Unique and Shared Aspects of Development. J. Mammary Gland Biol. Neoplasia.

[20] Sakakura T., Neville C.W., Daniel M.C. (1987). Mammary embryogenesis. The Mammary Gland: Development, Regulation, and Function.

[21] Rice R.H., Green H. (1979). Presence in human epithelial cells of a soluble protein precursor of the cross-linked envelope: Activation of the cross linking by calcium ions. Cell.

[22] Watt F.M., Green H. (1981). Involucrin synthesis is correlated with cell size in human epidermal cultures. J. Cell Biol..

[23] Schmid C., Zatloukal K., Beham A., Denk H. (1993). Involucrin expression in breast carcinomas: An immunohistochemical study. Virchows Arch..

[24] Tsuda H., Sakamaki C., Fukutomi T., Hirohashi S. (1997). Squamoid features and expression of involucrin in primary breast carcinoma associated with high histological grade, tumour cell necrosis and recurrence sites. Br. J. Cancer.

[25] Sun T.-T., Green H. (1976). Differentiation of the epidermal keratinocyte in cell culture: Formation of the cornified envelope. Cell.

[26] Watt F.M., Leigh M., Watt F.M. (1994). Keratinocyte Methods.

[27] Takahashi H., Ibe M., Kinouchi M., Ishida-Yamamoto A., Hashimoto Y., Iizuka H. (2003). Similarly potent action of 1,25-dihydroxyvitamin D3 and its analogues, tacalcitol, calcipotriol, and maxacalcitol on normal human keratinocyte proliferation and differentiation. J. Dermatol. Sci..

[28] Huang D.C., Papavasiliou V., Rhim J.S., Horst R.L., Kremer R. (2002). Targeted disruption of the 25-hydroxyvitamin D_3_ 1alpha-hydroxylase gene in ras-transformed keratinocytes demonstrates that locally produced 1alpha,25-dihydroxyvitamin D_3_ suppresses growth and induces differentiation in an autocrine fashion. Mol. Cancer Res..

[29] Bizarri M., Cucina A., Valente M.G., Taglaiferri F., Borrelli V., Stipa F., Cavallaro A. (2003). Melatonin and vitamin D_3_ increase TGF-beta1 release and induce growth inhibition in breast cancer cell cultures. J. Surg. Res..

[30] Finlay I.G., Stewart G.J., Ahkter J., Morris D.L. (2001). A phase one study of the hepatic arterial administration of 1,25-dihydroxyvitamin D_3_ for liver cancers. J. Gastroenterol. Hepatol..

[31] Dalhoff K., Dancey J., Astrup L., Skovsgaard T., Hamberg K.J., Lofts F.J., Rosmorduc O., Erlinger S., Hansen J.B., Steward W.P. (2003). A phase II study of the vitamin D analogue Seocalcitol in patients with inoperable hepatocellular carcinoma. Br. J. Cancer.

[32] Haussler M.R., Boyce D.W., Littledike E.T., Rasmussen H. (1971). A rapidly acting metabolite of vitamin D_3_. Proc. Natl. Acad. Sci. USA.

[33] Costa J.L., Eijk P.P., van de Weil M.A., ten Berge D., Schmitt F., Narvaez C.J., Welsh J., Ylstra B. (2009). Anti-proliferative action of vitamin D in MCF7 is still active after siRNA-VDR knock-down. BMC Genom..

[34] Hussain-Hakimjee E.A., Peng X., Mehta R.R., Mehta R.G. (2006). Growth inhibition of carcinogen-transformed MCF-12F breast epithelial cell and hormone sensitive BT-474 breast cancer cells by 1α-hydroxyvitamin D5. Carcinogenesis.

[35] Wang Q., Yang W., Uytingco M.S., Christakos S., Wieder R. (2000). 1,25-Dihydroxyvitamin D_3_ and all-trans-retinoic acid sensitize breast cancer cells to chemotherapy-induced cell death. Cancer Res.

[36] Jensen S.S., Madsen M.W., Lukas J., Binderup L., Bartek J. (2001). Inhibitory effects of 1alpha,25-dihydroxyvitamin D_3_ on the G(1)-S phase-controlling machinery. Mol. Endocrinol..

[37] Welsh J., Wietzke J.A., Zinser G.M., Byrne B., Smith K., Narvaez C.J. (2003). Vitamin D-3 Receptor as a Target for Breast Cancer Prevention. J. Nutr..

[38] Wang Q., Lee D., Sysounthone V., Chandraratna R.A.S., Christakos S., Korah R., Wieder R. (2001). 1,25-dihydroxyvitamin D_3_ and retinoic acid analogues induce differentiation in breast cancer cells with function- and cell-specific additive effects. Br. Cancer Res..

[39] Yang J., Ikezoe T., Nishioka C., Ni L., Koeffler H.P., Yokoyama A. (2010). Inhibition of mTORC1 by RAD001 (everolimus) potentiates the effects of 1,25-dihydroxyvitamin D_3_ to induce growth arrest and differentiation of AML cells in vitro and in vivo. Exp. Hematol..

[40] Kao J., Salari K., Bocanegra M., Choi Y.-L., Girard L., Ganghi J., Kwei K.A., Hernandez-boussard T., Wang P., Gazdar A.F. (2009). Molecular profiling of breast cancer cell lines defines relevant tumour models and provides a resource of cancer gene discovery. PLoS ONE.

[41] Hurst J., Maniar N., Tombarkiewicz J., Lucas F., Roberson C., Steplewski Z., James W., Perras J. (1993). A novel model of a metastatic human breast tumour xenograft line. Br. J. Cancer.

[42] Lopes N., Sousa B., Martins D., Gomes M., Vieira D., Veronese L.A., Milanezi F., Paredes J., Costa J.L., Schmitt F. (2010). Alternations in the vitamin D signalling and metabolic pathways in breast cancer progression: A study of VDR, CYP27B1, CYP24A1 expression in benign and malignant breast lesions Vitamin D pathways unbalanced in breast lesions. BMC Cancer.

[43] Pendás-Franco N., González-Sancho J.M., Suárez Y., Aguilera O., Steinmeyer A., Gamallo C., Berciano M.T., Lafarga M., Muñoz A. (2007). Vitamin D regulates the phenotype of human breast cancer cells. Differentiation.

[44] Neve R.M., Chin K., Fridlyand J., Yeh J., Baehner F.L., Fevr T., Clark L., Bayani N., Coppe J.-P., Tong F. (2006). A collection of breast cancer cell lines for the study of functionally distinct cancer subtypes. Cancer Cell.

[45] Harvey A.J., Crompton M.R. (2004). The Brk protein tyrosine kinase as a therapeutic target in cancer: Opportunities and challenges. Anti-Cancer Drugs.

[46] Alço G., Iğdem S., Dincer M., Ozmen V., Saglam S., Selamoglu D., Erdoğan Z., Ordu C., Pilancı K.N., Bozdogan A. (2014). Vitamin D Levels in Patients with Breast Cancer: Importance of Dressing Style. Asian Pac. J. Cancer Prev..

[47] Beer T.M., Ryan C.W., Venner P.M., Petrylak D.P., Chatta G.S., Ruether J.D., Redfern C.H., Fehrenbacher L., Saleh M.N., Waterhouse D.M. (2007). Double-Blinded Randomized Study of High-Dose Calcitriol Plus Docetaxel Compared With Placebo Plus Docetaxel in Androgen-Independent Prostate Cancer: A Report From the ASCENT Investigators. J. Clin. Oncol..

[48] Porter S., Scott S.D., Sassoon E.M., Williams M.R., Jones J.L., Girling A.C., Ball R.Y., Edwards D.R. (2004). Dysregulated expression of adamalysin-thrombospondin genes in human breast cancer. Clin. Cancer Res..

[49] Wall S.J., Edwards D.R. (2002). Quantitative reverse transcription-polymerase chain reaction (RT-PCR_: A comparison of primer-dropping, competitive, and real-time RT-PCRs. Anal. Biochem..

